# *Opuntia ficus‐indica* fruit consumption improves insulin resistance in mice with diet‐induced obesity

**DOI:** 10.1002/jsfa.70038

**Published:** 2025-07-25

**Authors:** Simona Terzo, Pasquale Calvi, Ignazio Restivo, Alessandro Massaro, Luisa Tesoriere, José‐Manuel Fernández‐Real, Marta Giardina, Antonella Amato, Flavia Mulè, Mario Allegra

**Affiliations:** ^1^ Department of Biological, Chemical and Pharmaceutical Sciences and Technologies (STEBICEF) University of Palermo Palermo Italy; ^2^ Department of Diabetes, Endocrinology and Nutrition Institut d'Investigació Biomèdica de Girona (IDIBGI) Girona Spain; ^3^ CIBERobn Fisiopatologia de la Obesidad y Nutrición; and Department of Medical Sciences, School of Medicine University of Girona Girona Spain

**Keywords:** *Opuntia ficus‐indica*, insulin resistance, functional food, HFD mice, gut microbiota

## Abstract

**BACKGROUND:**

Obesity induces various co‐morbidities including insulin resistance. *Opuntia ficus‐indica* fruit (OFIF) is rich in various beneficial compounds with antioxidant and anti‐inflammatory properties. The present study evaluated the efficacy of OFIF supplementation to counteract obesity‐induced insulin resistance in comparison with an euglycemic agent, chromium picolinate (CrPi), in high‐fat‐diet (HFD)‐fed mice. Molecular investigations associated with the observed effects as well as the evaluation of OFIF impact on the composition of gut microbiota in HFD mice were also carried out. Glucose metabolic parameters, oxidative stress and inflammation‐related biomarkers were analysed in liver and adipose tissue of HFD, HFD + OFIF and HFD + CrPI mice.

**RESULTS:**

OFIF supplementation, as well CrPi, improved glucose tolerance and insulin sensitivity (evaluated as HOMA‐IR) and increased hepatic and adipose tissue insulin receptor expression. Moreover, similar to CrPi, OFIF markedly decreased hepatic oxidative stress and inflammation in terms of NRF‐2, SOD‐2, NF‐kB, iNOS and COX‐2 protein expression levels. In adipose tissue, it reduced *IL‐1β*, *IL‐6*, *TNF‐α* gene expression and the density of crown‐like structures. In HFD mice, OFIF supplementation increased the presence of bacterial genera positively correlated to beneficial effects such as *Alloprevotella*, *Lachnospiraceae NK4A136 group* and *NK4A214_group*, while it reduced the abundance of some bacterial genera associated with harmful actions (*Staphylococcus*, *Romboutsia*, *Sporosarcina*, *Lachnoclostridium* and *Enterohabdus*).

**CONCLUSIONS:**

Our research demonstrates for the first time the euglycemic potential of OFIF and its ability to counteract obesity‐induced insulin resistance through not only its antioxidative and anti‐inflammatory properties but also its positive modulation of gut microbiota. © 2025 The Author(s). *Journal of the Science of Food and Agriculture* published by John Wiley & Sons Ltd on behalf of Society of Chemical Industry.

## INTRODUCTION

An emerging body of evidence highlights the relevance of functional foods for the prevention of those inflammation‐dependent, oxidative stress‐related conditions that can profoundly affect metabolic health.^1^, ^2^, ^3^, ^4^, ^5^, ^6^ *Opuntia ficus‐indica* (OFI), widely known for its pleasant‐tasting edible fruits and cladodes, is one of the most distributed fruit plants of arid and semi‐arid regions of the world. The health‐promoting properties of OFI plant cladodes and vegetative parts have been known for a long time.

Interestingly, more recent studies have also proposed OFI fruit (OFIF) as a source of valuable dietary components for human health.^7^, ^8^, ^9^, ^10^ OFIF exhibits a wide range of colours among different cultivars, spanning from green to white, yellow to orange and red to purple. These colour variations are primarily related to the combination of the betalain pigments present in the fruit, which also contains polyphenols, flavonoids, carotenoids, vitamins, minerals and amino acids.^7^, ^11^ All these bioactive compounds realise a peculiar and health‐promoting molecular signature that has repeatedly been reported to exert significant anti‐inflammatory and antioxidative effects both *in vivo* and *in vitro*.^8^, ^12^, ^13^, ^14^, ^15^

Over the last 20 years, the authors' research group has characterised the health benefits arising from the consumption of the yellow cultivar of OFIF that is specifically enriched in betaxanthins.^16^ Remarkably, nutritional studies conducted in healthy individuals have demonstrated that the consumption of OFIF positively influences the body's redox balance, by reducing lipid oxidative damage and enhancing the antioxidant status.^17^ Additionally, OFIF intake improves the inflammatory profile in healthy humans by lowering plasma levels of inflammatory markers such as TNF‐*α*, IL‐1*β*, IFN‐*γ*, IL‐8, C‐reactive protein and erythrocyte sedimentation rate, while increasing levels of the anti‐inflammatory cytokine IL‐10.^18^ These anti‐inflammatory effects were significantly associated with its antioxidative properties.

Metabolic syndrome (MetS) is a cluster of conditions, defined as the coexistence of at least three out of five risk factors, such as hyperglycaemia, hypertension, abdominal obesity, hypercholesterolaemia and reduced levels of high‐density lipoproteins.^19^, ^20^ MetS elevates the risk of cardiovascular diseases, type 2 diabetes and related morbidities. Its aetiology is largely attributed to the dysregulation of metabolic homeostasis associated with a systemic, low‐grade inflammation.^21^ In these conditions, visceral adipose tissue undergoes to a remodelling that leads to an increased release of basal fatty acids and pro‐inflammatory cytokines, hypoxia, fibrosis, decreased levels of adiponectin, insulin resistance (IR) and immune cell recruitment.^22^ Moreover, macrophages surround dead adipocytes, forming the so‐called crown‐like structures (CLSs).^23^ Interestingly, recent evidence also suggests that changes in composition and activities of gut microbiota may also play a crucial role in MetS development.^24^

IR can be defined as the reduced ability of an organism to mount a normal and coordinated glucose‐lowering response via tissue‐autonomous and crosstalk‐dependent mechanisms.^25^ This pathological condition results in a defective insulin signalling, strongly associated with a vicious cycle between inflammation and oxidative stress. IR occurs in a context of sub‐acute inflammation that originates from the influx of pro‐inflammatory cytokines, such as TNF‐*α* and IL‐6, released from adipocytes and Kupffer cells, as a result of adipose tissue and liver dysmetabolic activation. Within this scenario, nuclear factor kappa‐light‐chain‐enhancer of activated B cells (NF‐κB) plays a key role, by activating the synthesis of a complex system of pro‐inflammatory mediators and enzymes, such as IL‐1*β*, IL‐8, inducible nitric oxide synthase (iNOS) and cyclooxygenase 2 (COX‐2), that further exacerbate hepatic metabolic dysfunctions.^25^

Oxidative stress is also crucial in the development of IR. It results from the increase of reactive oxygen and nitrogen species (RONS) induced by mitochondrial dysfunction that is associated with enhanced and aberrant glucose and lipid catabolism.^26^, ^27^, ^28^, ^29^ Moreover, the concurrent downregulation in the expression levels of antioxidative enzymes further increases RONS levels, reinforcing overall oxidative stress. This downregulation strictly relies both on RONS overproduction and on the inhibition of nuclear erythroid 2‐related factor 2 (NRF‐2) activation. This protein is a key transcription factor that physiologically counteracts oxidative stress through the upregulation of crucial antioxidative defence systems such as heme oxygenase‐1 and superoxide dismutase‐2 (SOD‐2).^30^ However, when a chronic condition of excessive metabolic and oxy‐inflammatory stress is established, NRF‐2 activation is inhibited and/or downregulated.^31^, ^32^ Finally, increased levels of RONS can also perpetuate the pro‐inflammatory state, by further activating NF‐κB signalling in an aberrant and dysfunctional molecular crosstalk with NRF‐2.^33^, ^34^

In light of the intricate connections between inflammation, oxidative stress and IR, and considering the well‐documented antioxidative and anti‐inflammatory capacity of OFIF, we have here evaluated if OFIF supplementation could counteract the obesity‐induced, and oxy‐inflammation‐related, IR in an *in vivo* model of MetS. A possible impact of OFIF on the composition and abundance of gut microbiota was also considered. To this end, OFIF was administered to mice fed a high‐fat diet (HFD), which progressively develop a range of pathological alterations that resembles human MetS. Relevantly, euglycemic efficacy of OFIF was compared with that of a widely used supplement to ameliorate glycaemic dysmetabolism: chromium picolinate (CrPi).^35^, ^36^

A broad‐ranging evaluation of systemic, hepatic and adipose tissue dysmetabolic alterations as well as of oxy‐inflammation‐related signal transduction pathways and gut microbiota composition were performed to establish the efficacy of OFIF supplementation as a nutraceutical intervention for the management of MetS in comparison with CrPi.

## MATERIALS AND METHODS

### Animals and diet

A total of 32 males (4 weeks old) C57Bl/6j mice were supplied by Envigo Laboratories (San Pietro al Natisone, Udine, Italy). They were maintained in a 12 h light/dark cycle, at a temperature of 22 ± 2°C, with access to food and water *ad libitum*. Following a one‐week period of habituation, the mice were housed in acrylic cages with two animals per cage. The experiments were designed and carried out in accordance with the European guidelines for the production, care and use of laboratory animals. This project was reviewed and approved by the Ministry of Health (Rome, Italy; authorisation no. 37/2020‐PR on 16 January 2020).

A standard diet (4RF25, Mucedola, Milan, Italy) was provided to maintain the animals' health (*n* = 8, STD group) for 14 weeks, while a HFD (PF4051/D, Mucedola) comprising 60% kcal from fat for 10 weeks was used to induce obesity (*n* = 24). Diet composition and caloric content are given in detail in Table S1. Subsequently, two groups of obese mice (*n* = 8 per group) received twice‐daily oral administration of either 14 mg kg^−1^ lyophilised OFIF, prepared as previously reported^37^ (OFIF group), or 80 μg kg^−1^ CrPi (CrPi group) for 4 weeks. OFIF is characterised by a high percentage of moisture (>80%), minute amounts of proteins (13% of dry mass) and lipids, significant amount of sugars (10%), fibres (5%), vitamins (A, B, C, E), betalains and minerals (Ca^2+^, Mg^2+^, Mn^2+^, K^+^, Na^+^, P, Fe^2+^).^9^ The doses of OFIF and CrPi were chosen on the basis of the literature, because previously these doses were demonstrated to be efficacious against some aspects of MetS in obese^37^ or diabetic rats,^35^ respectively.

The integrations were dissolved in water and administered at a volume of 10 μL. The obese control group was fed a HFD for 14 weeks and received 10 μL of water to mimic the supplementation (HFD group, *n* = 8). The food intake and the body weight of the mice were recorded on a weekly basis throughout the study period. At the end of the experimental period, metabolic parameters were examined and then the animals were weighed and sacrificed by cervical dislocation. Plasma samples were prepared from blood collected from cardiac puncture just after the mice were killed and stored at −80 °C. The harvested liver and epididymal adipose tissue were rinsed with phosphate‐buffered saline (PBS), wiped with a paper towel, weighed and divided into two portions. One half was fixed in 4% formalin for histological assays, while the other was stored at −80 °C for biomolecular analyses.

### *In vivo* glucose tolerance and insulin tolerance tests

In overnight‐fasted mice (*n* = 8 per group), glucose tolerance test (GTT) and insulin tolerance test (ITT) were performed. For GTT, mice were injected intraperitoneally (i.p.) with glucose (2 g kg^−1^ body weight) while for ITT mice received i.p. insulin (0.5 U kg^−1^ body weight) (Lantus, Sanofi, Paris, France). Glucose levels were measured using a glucometer (GlucoMen LX meter, Menarini, Firenze) 0, 15, 30, 60 and 120 min after glucose or insulin injection in blood collected from the tail vein. The area under the curve (AUC) for both GTT and ITT was calculated using the trapezoidal method. The homeostatic model assessment for insulin resistance (HOMA‐IR) was calculated as [fasting insulin concentration (mU L^−1^) × fasting glucose concentration (mg dL^−1^) × 0.05551]/22.5.

### Plasma insulin measurement

Plasma insulin concentration was determined using a commercially available enzyme‐linked immunosorbent assay (ELISA) kit (Mercodia, Sylveniusgatan 8A, Uppsala, Sweden) according to the manufacturer's instructions.

### Immunoblotting analysis

Immunoblotting analyses were conducted as previously reported.^38^ Briefly, fractions of liver and adipose tissue were homogenised in ice‐cold lysis buffer containing 20 mmol L^−1^ Tris (pH 7.5), 137 mmol L^−1^ NaCl, 2 mmol L^−1^ EDTA, 1 mmol L^−1^ CaCl_2_, 1 mmol L^−1^ MgCl_2_, 1% NP‐40, 2 mmol L^−1^ sodium orthovanadate, 10 mmol L^−1^ sodium fluoride, 10 mmol L^−1^ sodium pyrophosphate, 10% glycerol, 2 μg mL^−1^ aprotinin, 5 μg mL^−1^ leupeptin and 2 mmol L^−1^ PMSF. The homogenates were then sonicated in ice‐cold conditions three times for 20 s and centrifuged at 12 000 rpm for 10 min at 4 °C. The supernatant was collected. The protein concentration was determined using a BCA assay kit. Equal amounts of proteins were used for western blot analysis. The proteins were heated at 95 °C for 5 min in sodium dodecylsulfate (SDS) sample loading buffer and separated using either 8% or 10% SDS–polyacrylamide gel electrophoresis (depending on the molecular weight of the proteins to be analysed) and transferred onto polyvinylidene fluoride membranes. After blocking with 5% non‐fat dry milk in Tris‐buffered saline with Tween 20 (TBST), membranes were incubated overnight at 4 °C with primary antibodies (1:1000 dilution), followed by a 1 h incubation at room temperature with horseradish peroxidase (HRP)‐linked secondary antibodies (anti‐rabbit IgG) in TBST. Visualisation was performed using a Bio‐Rad ChemiDoc™ imaging system. Band density of the target proteins was quantified after normalisation to *β*‐actin levels. Primary antibodies for insulin receptor (InsR), NRF‐2, NF‐*κ*B, COX‐2, iNOS, SOD‐2, *β*‐actin and HRP‐linked secondary antibody were obtained from Cell Signaling (Danvers, MA, USA).

### Malondialdehyde (MDA) analysis in liver and hepatic tissues

The MDA levels were assessed using the thiobarbituric acid (TBA) assay. Approximately 30 mg of tissue (liver or adipose) was homogenised in 300 μL of ice‐cold PBS. The TBA reaction was carried out following the manufacturer's instructions. Briefly, samples were incubated with TBA and SDS at 95 °C for 1 h, followed by centrifugation at 800 × *g* for 10 min. The supernatants were then transferred to a 96‐well plate, and absorbance was measured at 532 nm. Protein concentration in each sample was quantified using a BCA assay kit (Merck, Milan, Italy). MDA concentration was calculated using the formula provided by the manufacturer: MDA (nmol mL^−1^) = (sample OD value − background OD)/(standard OD − blank OD) × standard concentration (10 nmol mL^−1^) × sample dilution factor. After calculation, tissue MDA levels were further normalised to the protein concentration of each sample (mg protein mL^−1^). MDA levels were expressed as nmol of MDA per mg of tissue.

### RONS analysis in liver and hepatic tissues

The levels of RONS in the liver and adipose tissues were measured following a standard procedure based on the oxidation of 2′,7′‐dichlorodihydrofluorescin diacetate (DCFH‐DA; Merck, Milan, Italy) to 2,7‐dichlorofluorescein (DCF). In brief, the reaction mixture included 150 μL of 0.1 mol L^−1^M potassium phosphate buffer (pH 7.4), 10 μL of tissue homogenate, 35 μL of distilled water and 5 μL of DCFH‐DA solution (final concentration of 5 μmol L^−1^). Fluorescence resulting from the oxidation of DCFH‐DA was recorded over 10 min, with measurements taken every 30 s at excitation and emission wavelengths of 488 and 525 nm, respectively. The amount of DCF formed was quantified in arbitrary units.

### Immunohistochemistry

Visceral white adipose tissue was dissected and preserved in a 4% formaldehyde solution. After fixation, the tissue was embedded in paraffin and sectioned at 5 μm thickness. The paraffin‐embedded adipose tissue sections were deparaffinised, followed by treatment with 3% hydrogen peroxide (H₂O₂) to inhibit endogenous peroxidase activity. After rinsing with PBS, the sections were incubated with a 3% normal serum blocking solution in PBS for 30 min. The samples were then incubated overnight at 4 °C with a monoclonal anti‐MAC‐2 antibody (diluted 1:1500; Cedarlane Laboratories, Burlington, Ontario, Canada), which identifies activated macrophages. Following a thorough PBS wash, the sections were incubated for 30 min with a 1:300 dilution of anti‐mouse IgG biotinylated HRP‐conjugated secondary antibody (Vector Laboratories, Burlingame, CA, USA) in PBS. The histochemical staining was carried out using 3,3′‐diaminobenzidine (Sigma‐Aldrich, Vienna, Austria) as the substrate. After counterstaining with haematoxylin, the sections were dehydrated in ethanol and mounted using Eukitt® Mounting Medium (Sigma‐Aldrich, Merck KGaA, Darmstadt, Germany). The tissue sections were examined using a light microscope (Leica DMLB, Meyer Instruments, Houston, TX, USA) equipped with a DS‐Fi1 camera (Nikon, Florence, Italy), and digital images were captured at a magnification of ×10. The density of CLSs was quantified by counting the total number of CLSs per section, normalising it to the total number of adipocytes and expressing it as CLSs per 10 000 adipocytes.

### Reverse transcription PCR analysis

Total RNA was extracted from adipose tissue using the TRIzol reagent (Invitrogen, Thermo Fisher Scientific, Waltham, MA, USA), and complementary DNA (cDNA) was synthesised from 2 ng of total RNA using a High‐Capacity cDNA Reverse Transcription Kit (Applied Biosystems, Waltham, MA, USA). The cDNA (2.5 μL) was then amplified with specific forward and reverse primers. For the detection of TNF‐*α*, IL‐1*β* and IL‐6, 40 amplification cycles were performed (15 s at 95 °C, 15 s at 63 °C and 15 s at 72 °C) using the following primers: for TNF‐*α*, forward primer 5′‐AGCCCACGTCGTAGCAAACCA‐3′ and reverse primer 5′‐GCAGGGGCTCTTGACGGCAG‐3′; for IL‐1*β*, forward primer 5′‐TGCCACCTTTTGACAGTGATG‐3′ and reverse primer 5′‐GGAGCCTGTAGTGCAGTTGT‐3′; and for IL‐6, forward primer 5′‐GCCTTCTTGGGACTGATGCT‐3′ and reverse primer 5′‐TGTGACTCCAGCTTATCTCTTGG‐3′. For *β*‐actin detection, 40 amplification cycles were conducted (15 s at 95 °C, 15 s at 60 °C and 15 s at 72 °C) using the primers 5′‐CGGGATCCCCGCCCTAGGCACCAGGGT‐3′ and 5′‐GGAATTCGGCTGGGGTGTTGAAGGTCTCAAA‐3′. PCR products were separated on a 1.8% agarose gel and visualised under UV light.

### Gut microbiota composition

Faecal samples were collected from animals and stored at −80 °C until further processing. DNA was extracted using a QIAamp DNA Stool Handbook Kit (Qiagen, Hilden, Germany), following the protocol provided by the manufacturer. Eight replicates were processed for STD‐ HFD‐, OFIF and CrPi groups. The extracted DNA served as the basis for a metagenomic analysis conducted by BMR Genomics (Padova, Italy).

For next‐generation sequencing, PCR amplification was performed, as previously described.^39^ The amplified PCR products were purified using Agencourt XP 1X magnetic beads (Thermo Fisher Scientific, MA, USA) and directly processed for sequencing library preparation on the Illumina MiSeq platform (San Diego, CA, USA). Taxonomic annotations were performed using the UCLUST algorithm (QIIME 2) to classify sequences, with a minimum identity threshold of 97%, against the Greengenes v13.8 reference database (https://docs.qiime2.org/2022.2/data-resources/). Both seed and unmatched nonseed sequences were annotated. Operational taxonomic units (OTUs) were extracted from the resulting biom file and filtered at an abundance threshold of 0.005% to remove low‐frequency, spurious OTUs. This ensured the inclusion of only biologically meaningful taxa in subsequent analyses. Alpha diversity analyses were performed on all samples using the observed features, Shannon entropy and Pielou evenness.

### Statistical analysis

Statistical analyses were conducted using GraphPad Prism (GraphPad Software, Inc., La Jolla, CA, USA). Data are presented as mean ± standard error of the mean (SEM). Significant differences between groups were assessed using one‐way analysis of variance followed by Bonferroni *post hoc* tests. A *P* value of less than 0.05 was considered statistically significant.

The identification of microbial genera associated with OFIF or CrPi treatment was performed using the method of compositional microbiome analysis with bias correction (ANCOM‐BC)^40^ implemented in R software (version 4.4.1). ANCOM‐BC accounts for bias due to different sampling fractions between samples by incorporating a sample‐specific offset into a linear regression model derived from the observed data. To account for the compositional nature of metagenomic datasets, the log‐scaled linear regression model is equivalent to a log‐likelihood transformation, with the offset term acting as a bias correction. Results were adjusted by treatment group to ensure that the analysis accounted for treatment‐specific differences. Statistical significance was set at *P* < 0.1.

The relationships between oxidative stress, inflammation, glucose dysmetabolism markers and bacterial genera were evaluated using Spearman correlations. Significance of Spearman correlation coefficients was adjusted by the Benjamini–Hochberg method (false discovery rate). *P* < 0.1 was considered statistically significant. A heatmap was used to show the correlation analysis.

## RESULTS

### Effects of OFIF on body weight and food consumption

**Figure 1:**
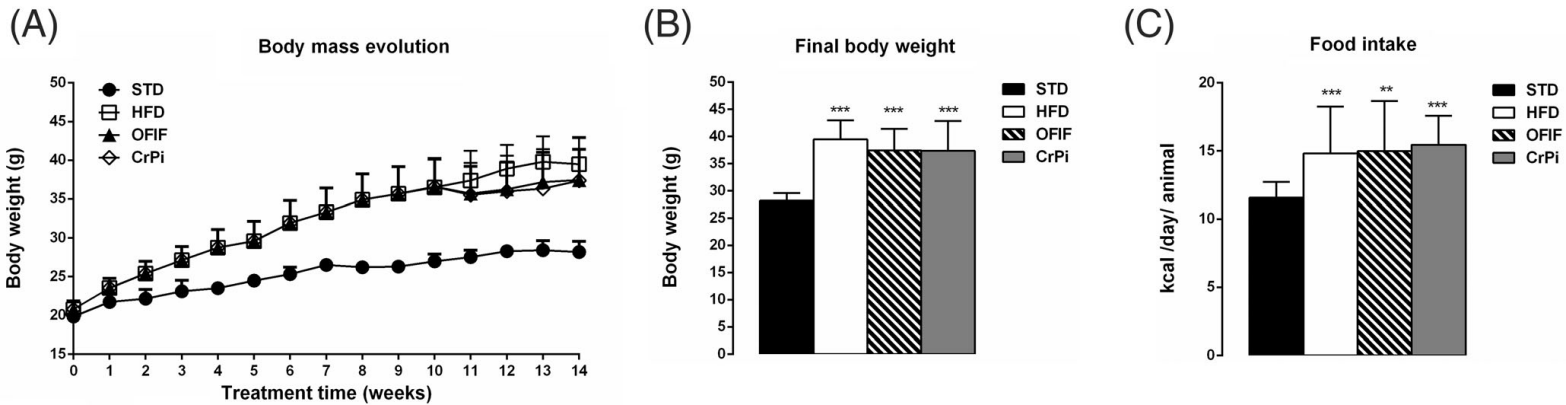
Changes in body weight and food intake in different groups of animals. (A) Temporal evolution of body weight, (B) final body weight and (C) food intake (kcal per day per animal) in mice fed STD, HFD, OFIF and CrPi. Data are mean ± SEM (*n* = 8 per group). **P < 0.01, ***P < 0.001 *versus* STD mice.

The data concerning body weight and food intake in the different groups of mice are shown in Fig. 1. The temporal evolution of body weight (Fig. 1(A)) showed an increase in all groups from the beginning of the experimentation until the end of the experimental protocol (week 14). To be noted is a slight reduction trend during the supplementation weeks in OFIF or CrPi mice. The final body weights of mice fed HFD or HFD plus OFIF or HFD plus CrPi were significantly higher than those of STD mice (Fig. 1(B)). However, OFIF and CrPi treatment did not prevent body weight gain.

The food intake (kcal per day per animal) was significantly higher in HFD, OFIF and CrPi groups compared to the STD group (Fig. 1(C)), without any significant differences among the obese animal groups.

### Effect of OFIF on HFD‐induced glucose intolerance and IR

**Figure 2:**
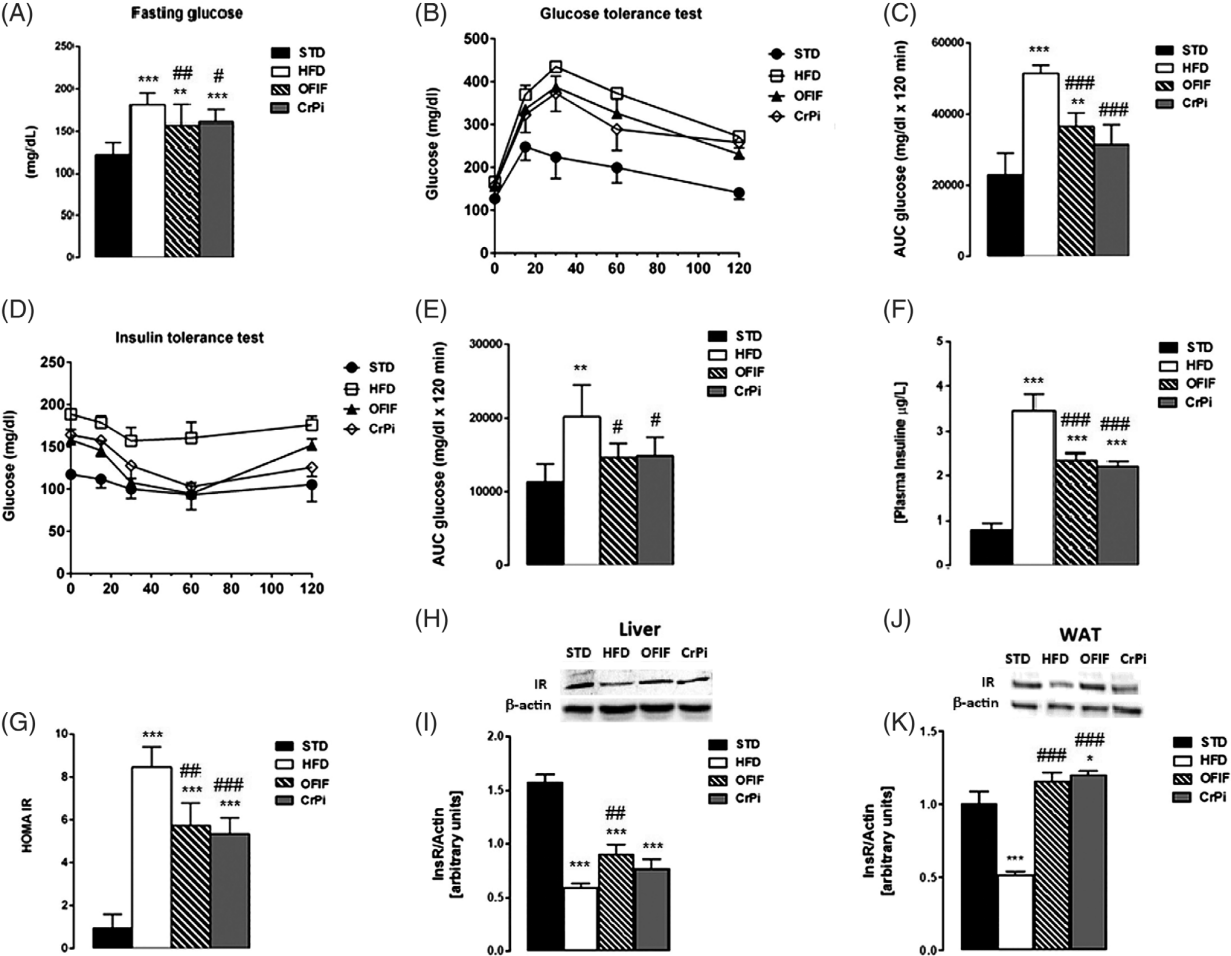
Changes in glucose metabolism parameters in different groups of animals. (A) Fasting blood glucose, (B) GTT, (C) AUC of GTT, (D) ITT, (E) AUC of ITT, (F) plasma insulin concentration, (G) IR index. (H, I) expression levels of insulin receptor in the liver and (J, K) in epididymal adipose tissue. Representative results of Western blot analysis are shown above each bar plot, and quantitative analysis of the proteins is shown below the blots. Data are mean ± SEM (*n* = 8 per group). *P < 0.05, **P < 0.01, ***P < 0.001 *versus* STD mice; #*P* < 0.05, ##*P* < 0.01 *versus* HFD mice; ###*P* < 0.001 *versus* HFD mice.

Fasting blood glucose was significantly lower in OFIF and CrPi groups than HFD group (Fig. 2(A)). The GTT assay and calculated AUC showed that both OFIF and CrPi improved glucose clearance (Fig. 4(B),(C)). In the ITT assay, OFIF and CrPi mice were more sensitive to insulin than HFD mice (Fig. 2(D),(E)). The plasma insulin levels of mice in OFIF and CrPi groups were lower than those in the HFD group (Fig. 2(F)). HOMA‐IR was higher in HFD mice than in STD, OFIF and CrPi mice (Fig. 4(G)), suggesting an improvement by OFIF of glucose homeostasis. As expected, quantitative analysis revealed a significant reduction in the expression levels of InsR in liver and epididymal adipose tissue of HFD mice, in comparison with those in the STD group. Remarkably, OFIF treatment significantly increased InsR expression in the liver with respect to the HFD group and restored its levels to control values in adipose tissue. Interestingly, while CrPi was not effective in increasing InsR protein levels in the liver with respect to the HFD group, it increased these levels above control values in adipose tissue (Fig. 2(H)–(M)).

### Effects of OFIF on hepatic and adipose tissue oxidative stress

**Figure 3:**
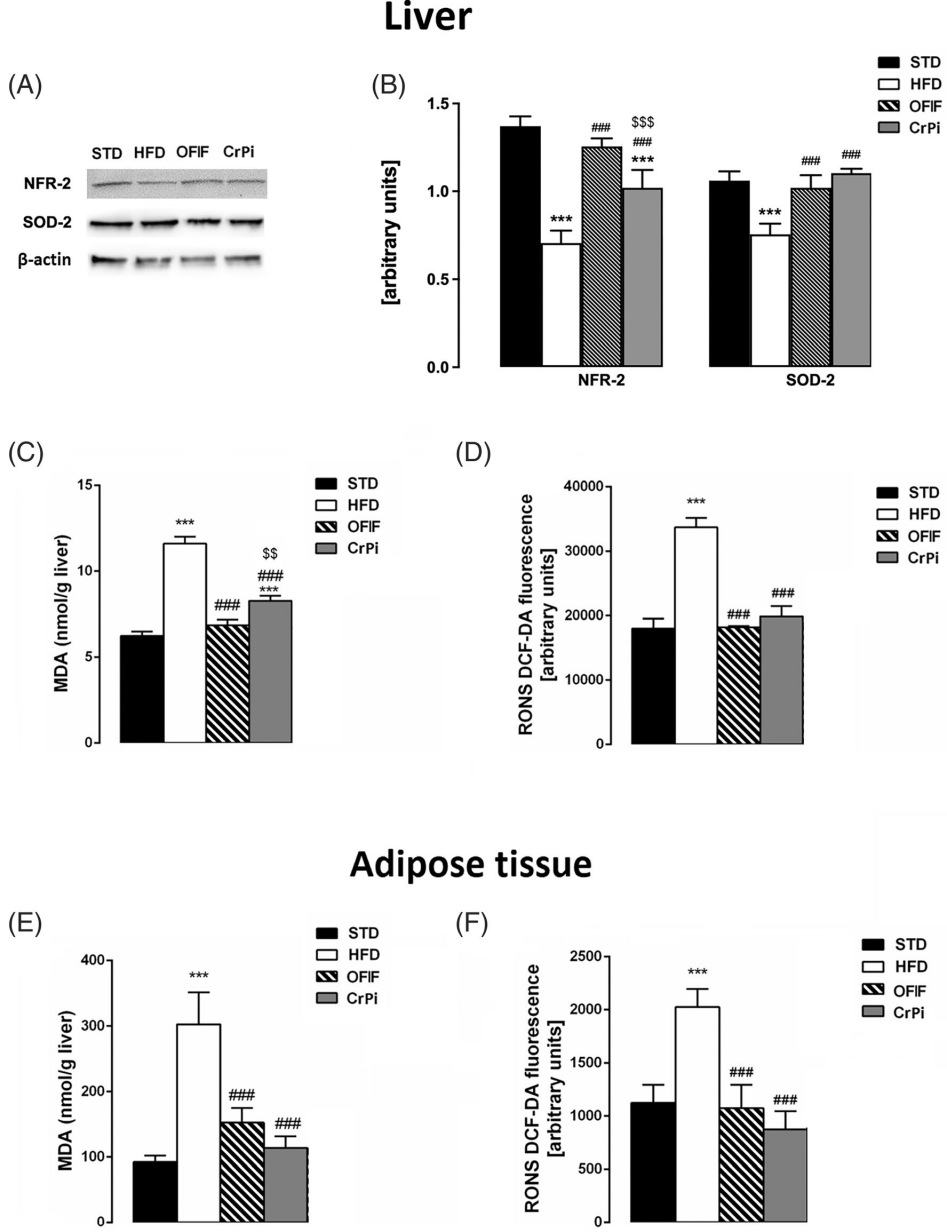
Evaluation of liver and adipose tissue oxidative stress in different groups of animals. (A, B) Hepatic protein expression levels of NRF‐2 and SOD‐2. (A) Representative results of Western blot analysis and (B) quantitative analysis of the proteins. (C) Liver MDA and (D) RONS levels. (E) Adipose tissue MDA and (F) RONS levels. Data are mean ± SEM (*n* = 8 per group). ***P < 0.001 *versus* STD mice; ###*P* < 0.001 *versus* HFD mice; $$*P* < 0.01; $$$*P* < 0.001 *versus* OFIF mice.

Oxidative stress plays an important role in the development of obesity‐related co‐morbidities such as IR. Therefore, we next evaluated the effects of OFIF on the expression levels of NRF‐2, a key transcription factor involved in the endogenous antioxidative defence system. As shown in Fig. 3, the protein expression levels of NRF‐2 in the liver were significantly lower in HFD mice in comparison with those in STD mice. Remarkably, OFIF administration increased NRF‐2 levels to control values with a significantly higher efficacy compared to CrPi (Fig. 3(A),(B)). Coherently, the expression levels of SOD‐2 were significantly decreased in HFD mice with respect to STD mice, and OFIF administration or CrPi restored its levels to control values (Fig. 3(A),(B)). To further evaluate the efficacy of OFIF to counteract liver and adipose tissue oxidative stress, we then assessed the levels of two of the key markers involved in tissue oxidative stress, i.e. MDA and RONS. Remarkably, our results showed that the levels of MDA and RONS that were significantly increased in both liver and adipose tissues of HFD mice were restored to control values in OFIF and CrPi mice (Fig. 3(C)–(F)).

### OFIF effects on hepatic and adipose tissue inflammation

**Figure 4:**
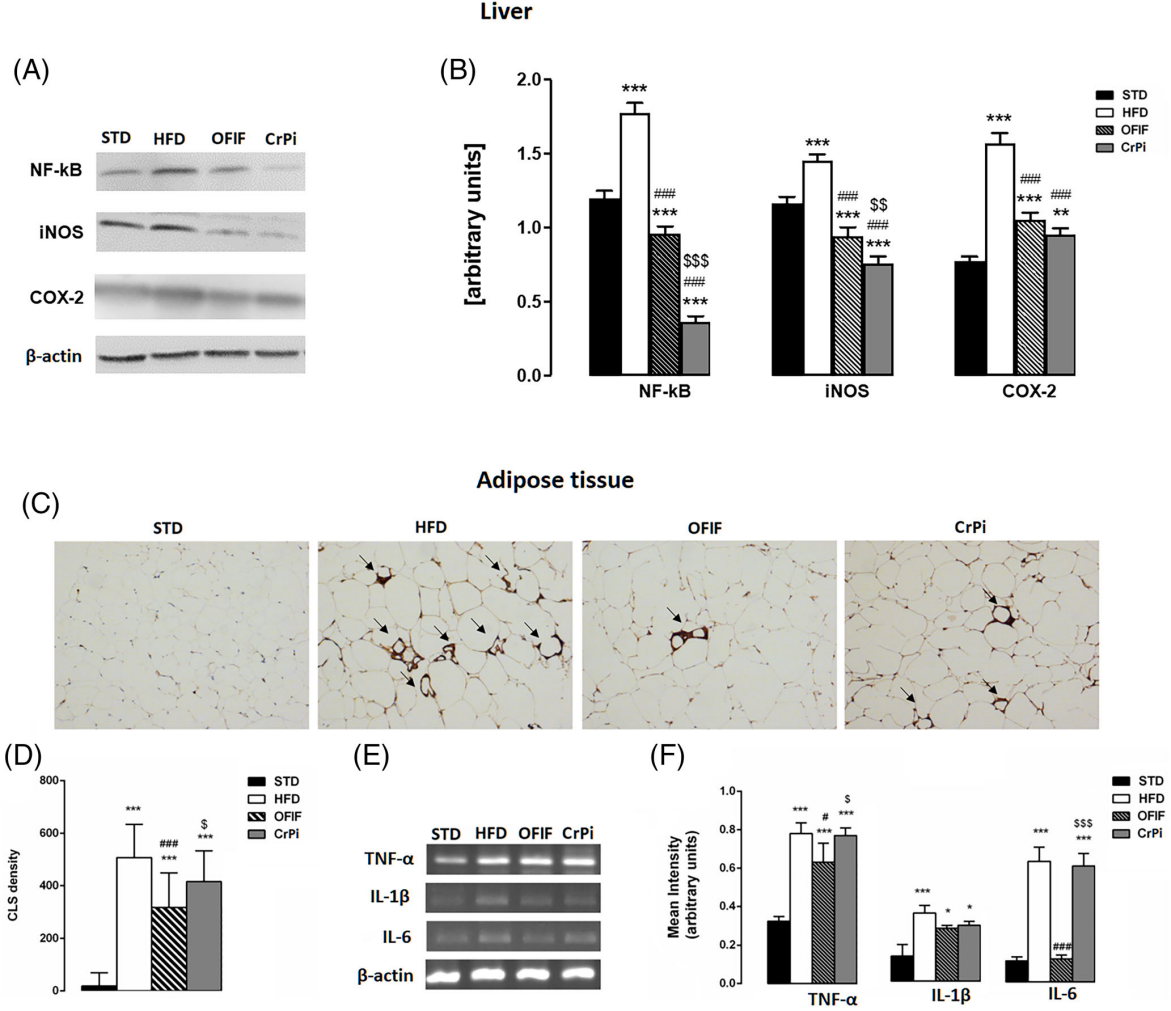
Evaluation of hepatic and adipose tissue inflammation in different groups of animals. (A) Representative results of Western blot analysis and (B) quantitative analysis of NF‐*κ*B, iNOS and COX‐2 protein expression levels in the liver. (C) Immunostaining with Mac‐2 antibody (brown) in epididymal adipose tissue sections. (D) Quantification of CLS density of the immunostained section and (E) mRNA levels of the proinflammatory genes *TNF‐α*, *IL‐1β* and *IL‐6*. (F) Results of semiquantitative PCR. Data are mean ± SEM (*n* = 8 per group). *P < 0.05, **P < 0.01, ***P < 0.001 *versus* STD mice; #*P* < 0.5, ###*P* < 0.001 *versus* HFD mice; $*P* < 0.5, $$*P* < 0.01, $$$*P* < 0.001 *versus* OFIF mice.

We next evaluated the efficacy of OFIF supplementation to ameliorate hepatic, HFD‐induced inflammation in comparison with CrPi. As shown in Fig. 4((A),(B)), the HFD regimen significantly promoted NF‐*κ*B overexpression and, coherently, increased protein expression levels of both COX‐2 and iNOS. Remarkably, OFIF and CrPi were able to reduce NF‐*κ*B overexpression, restoring the levels below control values. In line with these evidences, iNOS protein expression levels were significantly reduced even below control values by OFIF or CrPi. COX‐2 levels also were significantly reduced by OFIF or CrPi. Finally, the presence of Mac‐2, index of inflammation, was assessed by immunohistochemistry in adipose tissue of the different groups of animals. The results showed that the CLS density was reduced in OFIF and CrPi groups compared to the HFD group (Fig. 4(C)). However, as shown in Fig. 4(D), CLS number was significantly reduced only in OFIF mice.

Accordingly, gene expression of *TNF‐α*, *IL‐1β* and *IL‐6* was reduced in adipose tissue of OFIF animals in comparison with the HFD group (Fig. 4(E),(F)).

### Effect of OFIF on gut microbiota composition

Dysbiosis has been associated with obesity‐related dysfunctions, such as IR and MetS.^41^ Therefore, we investigated the impact of OFIF on composition and abundance of gut microbiota.

**Figure 5:**
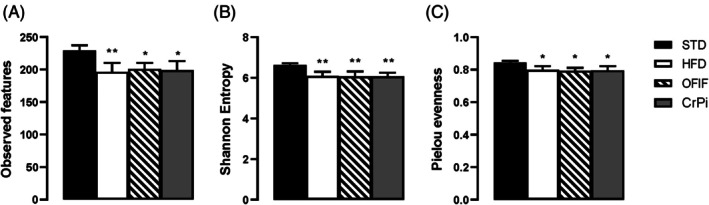
Alpha diversity of gut microbiota in different animal groups evaluated by (A) observed features, (B) Shannon entropy and (C) Pielou evenness. Data are mean ± SEM (*n* = 8 per group). *P < 0.05, **P < 0.01 *versus* STD mice.

In HFD mice, alpha diversity indices, namely observed features, Shannon entropy and Pielou evenness, were significantly lower compared to STD mice. However, no significant difference was detected between HFD and OFIF or CrPi groups (Fig. 5).

**Figure 6:**
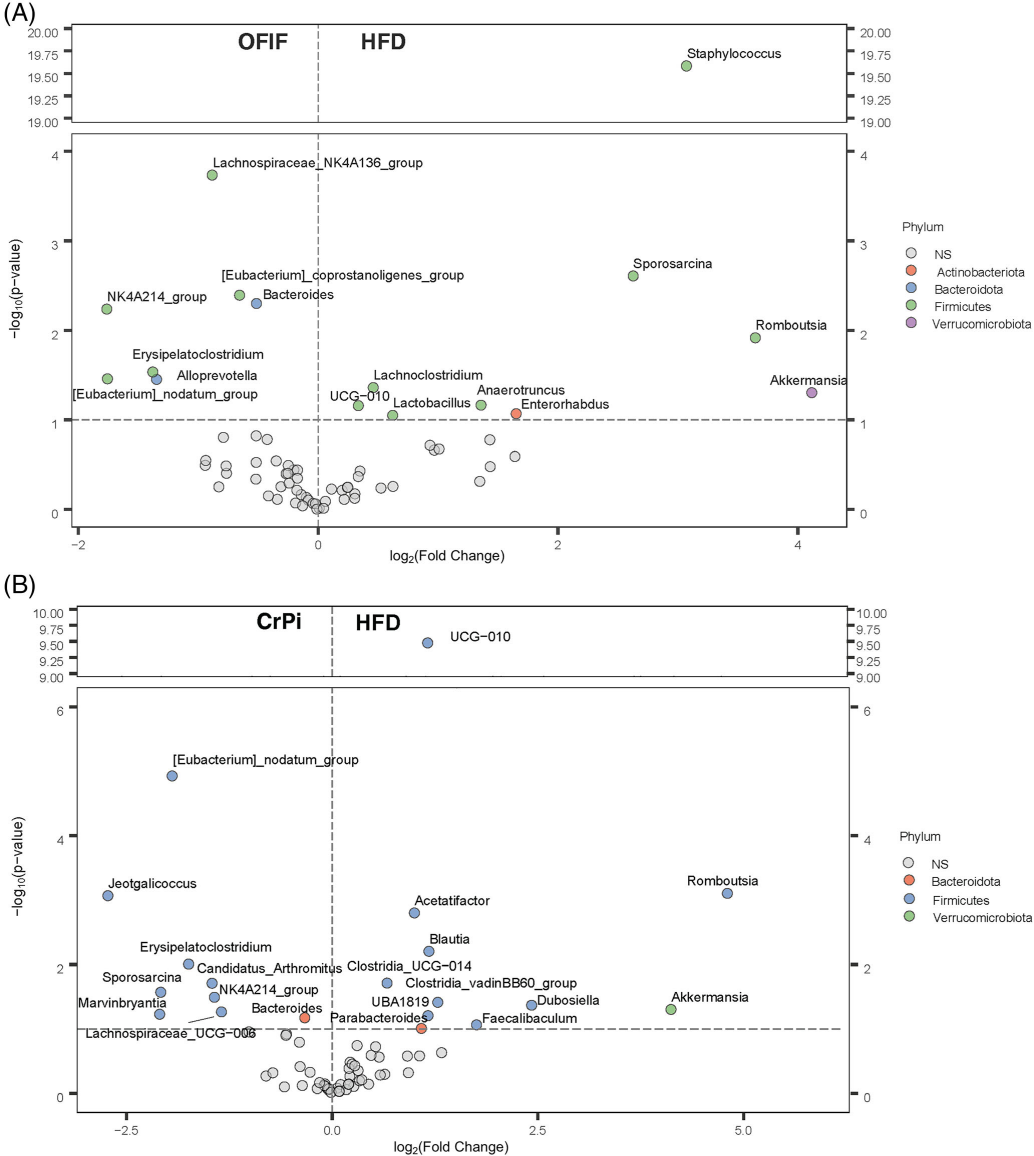
Volcano plot of differential microbial abundance between OFIF mice and HFD mice. Significantly different genera are coloured according to phylum. Significance was set at *P* < 0.1.

Moreover, we performed ANCOM‐BC through R packages to reveal bacterial genera that were differentially abundant among the different groups of mice. As expected, numerous bacterial genera were found to be differentially abundant between the HFD and STD mice (Fig. S1, supporting information). The same analysis revealed differentially abundant bacterial genera between the HFD group and the OFIF group. *Alloprevotella*, *Lachnospiraceae NK4A136_group* and *NK4A214_group* were among the genera that were more abundant in the OFIF group compared to the HFD group (Fig. 6(A)). In addition, abundance of *Staphylococcus*, *Romboutsia*, *Sporosarcina*, *Lachnoclostridium* and *Enterohabdus* was increased in the HFD group, implying that these species were reduced in the HFD + OFIF group (Fig. 6(A)). The analysis between HFD and CrPi groups revealed that the genera *Candidatus Arthromitus*, *Marvinbrvantia*, *Lachnospiraceae_UCG‐006* and *NK4A214_group* were significantly dominant in the CrPi group (Fig. 6(B)). On the other hand the abundance of the genera *Romboutsia*, *Acetatifactor* and *Blautia* was increased in the HFD group, implying that these species were reduced in the HFD + OFIF group.

**Figure 7:**
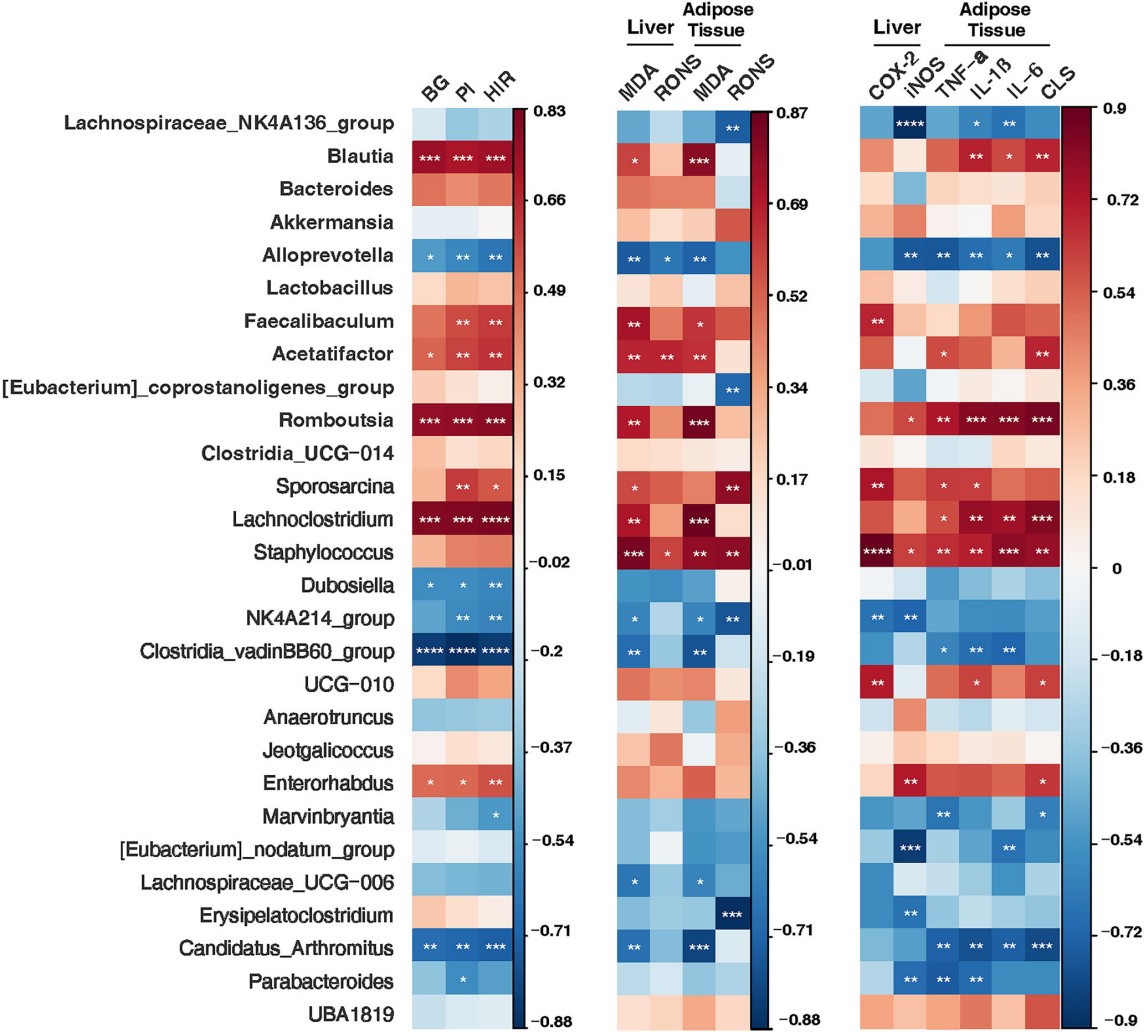
Heatmap displaying the Spearman correlation between microbiota genera differently abundant after OFIF treatment in comparison with HFD group and glucose dysmetabolism, oxidative stress and inflammatory markers. The colours range from red (positive correlation) to blue (negative correlation). BG, blood fasting glucose concentration; PI, plasma insulin level; HIR, HOMA, index of insulin resistance. Significant associations are shown with asterisks: ****P < 0.001, ***P < 0.01, **P < 0.05, *P < 0.1 adjusting by false discovery rate.

We evaluated the possible link among the changes in the composition of bacterial genera and the glucose metabolism parameters, as well oxidative stress and inflammation parameters altered in HFD mice through Spearman correlation analysis (Fig. 7). The analysis showed that bacterial genera *Alloprevotella*, *NK4A214_group* and *Candidatus Arthromitus* were negatively correlated with the indexes of glucose dysmetabolism, particularly HOMA index, and the markers of oxidative stress and inflammation. *Lachnospiraceae_NK4A136_group*, *[Eubacterium]_nodatum_group* and *Marvinbryantia* mainly were negatively correlated to inflammation markers. On the contrary, abundances of *Romboutsia*, *Lachnoclostridium*, *Sporosarcina*, *Blatia* and *Acetatifactor* were positively correlated with almost all indexes of glucose dysmetabolism, oxidative stress and inflammation that we considered. Moreover, a positive association was found between *Enterorhabdus* presence and glucose dysmetabolism parameters, while *Staphylococcus* was positively correlated with oxidative stress and inflammation markers (Fig. 7).

## DISCUSSION

This study assessed the effectiveness of OFIF supplementation in mitigating obesity‐induced, oxy‐inflammation‐related IR and its potential impact on gut microbiota in an *in vivo* model of MetS, specifically HFD mice. A key aspect of the research is the selected OFIF dose (14 mg kg^−1^), previously shown to be efficacious in preventing some aspects of MetS in HFD rats^37^ and chosen with the intent of being applicable as a nutraceutical supplement for humans. Notably, this dosage, when administered to a 75 kg individual, could be conveniently encapsulated into two nutraceutical capsules (1050 mg). Furthermore, the study was designed to compare the bioactivity of OFIF with CrPi, a widely used food supplement for managing dysglycaemic conditions.

As previously mentioned, the HFD model is considered highly translational as it mimics obesity in humans, readily inducing weight gain and increased fat mass.^42^ In this regard, in our experimental conditions, while HFD significantly increased both the body weight and the food intake in comparison to the STD regimen, neither OFIF nor CrPi were able to reduce significantly these parameters. These results appear in contrast with those previously published in a recent work by the author's research group^37^ and might be related to the differences in the two experimental setups. Indeed, while in the paper by Gambino *et al*.^37^ rats were used and MetS induction was achieved in 8 weeks, the present work employed mice and the HFD regimen was prolonged for 10 weeks, in accordance with other previously published works.^38^ The different types of animals employed, and the more intense induction of MetS might account for the currently observed inefficacy of OFIF to induce a significant reduction of both parameters, inducing just a negative trend.

The main focus of our research was to evaluate the efficacy of OFIF in ameliorating HFD‐induced IR and to analyse the underlying mechanisms. Along these lines, remarkable evidence is the ability of OFIF to improve all the major plasma glycaemic parameters, including fasting glycaemia and insulinemia, HOMA‐IR, glucose tolerance and insulin response, in comparison to the HDF mice. To the best of our knowledge, this is the first report giving evidence of the antihyperglycemic efficacy of OFIF in an *in vivo* model of HFD‐induced IR. Indeed, all the available scientific data on the ability of the OFI plant to counteract IR are related to other vegetative parts such as cladodes, seeds and skin that have a completely different bioactive fingerprint.^11^ Moreover, no evidence on the euglycemic effects of the yellow cultivar of the plant has so far been reported.

Another relevant aspect of the current study has been to compare OFIF euglycemic efficacy to that of CrPi, a diffused food supplement, widely investigated for the control of dysglycaemic states and for the prevention of IR.^35^, ^36^ In this regard, in our model OFIF showed the same efficacy as CrPi in the improvement of glucose and insulin tolerance and HOMA‐IR, suggesting beneficial effects on glucose metabolism comparable to those exerted by CrPi. At a molecular level, OFIF and CrPi show the same efficacy in counteracting HFD‐induced InsR downregulation, restoring the levels of its protein expression to control values in adipose tissue (CrPi even above) but not in the liver. The different mechanisms underlying InsR downregulation in these two metabolic areas^43^ might well account for the reduced effectiveness of the different supplementations between liver and adipose tissue.

As previously stated, oxidative stress plays a major role in the aetiology of obesity‐related IR, being a causative agent in the development of type 2 diabetes and all its associated complications.^29^, ^44^ Within this scenario, the ability of OFIF to counteract IR, ameliorating the glucidic profile in our model of MetS, could also result from its antioxidative potential.^17^, ^18^ In line with this hypothesis, our results clearly show that OFIF administration markedly decreased levels of oxidative stress markers, such as MDA and RONS in liver and adipose tissue, restoring them to control values. Interestingly, these results are consistent with the ability of OFIF to reduce the HFD‐induced increase of lipid peroxidation also in the brain.^37^ Remarkably, our data also underlie how the antioxidative effects of OFIF are overall comparable to those exerted by CrPi.

From a mechanistic perspective, the molecular events sustaining the OFIF antioxidative effects have also been taken into account in the liver. Indeed, our results clearly show how the amelioration of oxidative unbalance by OFIF is associated with the improvement of the tissue antioxidative defence system, in terms of both NRF‐2 and SOD‐2 expression levels. Relevantly, while the NRF‐2 modulating effects of CrPi are well documented,^45^, ^46^, ^47^ this is the first report showing, *in vivo*, the ability of OFIF to interfere with the NRF‐2 molecular axis, fostering liver antioxidative response, during MetS conditions. Within this scenario, the antioxidative and bioactive profile of OFIF, containing betalains, polyphenols, flavonoids, carotenoids, vitamins, minerals and amino acids, might play a key role in the modulation of redox homodynamics, whose alteration strongly sustains obesity‐related IR.

Development of obesity‐driven IR strictly relies on liver and adipose tissue inflammation. As previously described, this condition is generated by the persistent recruitment and infiltration of macrophages that orchestrate the elaboration of a complex and interconnected network of pro‐inflammatory mediators. Our results, showing the ability of OFIF to reduce the levels of two of the major liver pro‐inflammatory enzymes, i.e. iNOS and COX‐2, appear of relevance, especially when integrated with those obtained for adipose tissue. Here, OFIF administration reduced *IL‐1β*, *IL‐6*, *TNF‐α* gene expression and the density of CLSs. As a whole, these findings demonstrate for the first time, in an *in vivo* context, the ability of OFIF to counteract liver and adipose tissue inflammation through the reduction of macrophage activation and infiltration. Remarkably, in our conditions, these results are associated with the inhibition of NF‐κB overexpression in the liver. Together with the currently observed modulation of NRF‐2 expression, they provide an integrated picture of two key molecular events underlying the anti‐inflammatory and redox‐modulating properties of OFIF, relevant to explain the euglycemic potential of the fruit. Moreover, these results align with previously published data from our group, demonstrating that OFIF consumption exerts significant anti‐inflammatory effects in healthy humans.^18^

The gut microbiota is critical for homeostasis and control of metabolic disorders.^48^ Evidence suggests that HFD negatively affects bacterial composition, reducing bacteria associated with beneficial effects and increasing bacteria with harmful effects.^49^ In consideration of the pivotal functions of the gut microbiota in metabolic diseases, we verified whether the antihyperglycemic effects of OFIF may be partially attributable also to the modulation of the gut microbiota.

The results of the present study confirmed the strong impact of HFD on gut microbiota composition; however, OFIF as well as CrPi supplementation did not significantly alter the HFD‐induced dysbiosis as evidenced by alpha diversity analysis. It is noteworthy that OFIF treatment increased the presence of some bacterial genera, resulting in our Spearman correlation analysis being associated with beneficial effects such as *Alloprevotella*, *Lachnospiraceae NK4A136 group* and *NK4A214_group*, while it reduced the abundance of some bacterial genera associated with harmful actions (*Staphylococcus*, *Romboutsia*, *Sporosarcina*, *Lachnoclostridium* and *Enterohabdus*) in comparison with HFD mice. Within this scenario, the beneficial effects of OFIF could be also attributable, at least in part, to the changes in the composition and activity of the gut microbiota. To note is that CrPi treatment altered the abundance of some bacterial genera that were affected also by OFIF treatment (*NK4A214_group* and *Romboutsia*). Additionally, it increased the relative abundance of other bacterial genera that, according to our Spearman analysis, were associated with positive effects, including *Candidatus Arthromitus*, *Marvinbryantia* and *Lachnospiraceae_UCG‐006*. CrPi also reduced the abundance of bacterial genera linked to potentially harmful actions, such as *Acetatifactor* and *Blautia*.

In particular, Spearman correlation analysis showed that *Alloprevotella*, whose abundance was increased by OFIF supplementation, was negatively correlated with the glucose dysmetabolism parameters and the markers of oxidative stress and inflammation that we considered, suggesting a possible involvement of *Alloprevotella* in euglycemic effects of OFIF. Consistent with our results, *Alloprevotella* has been reported to promote anti‐inflammatory effects^50^ and it has been associated with beneficial effects.^51^ Similarly, *Lachnospiraceae NK4A136 group* and *NK4A214_group*, that belongs to the Oscillospiraceae family, were negatively correlated with adipose tissue RONS and inflammatory markers. *Lachnospiraceae NK4A136 group* and the Oscillospiraceae family are important producers of butyrate, a compound with well‐documented beneficial effects, because it supports intestinal barrier function, inhibits inflammation^52^ and enhances the survival of pancreatic *β*‐cells.^53^ Since these bacterial genera were increased by OFIF supplementation, they could contribute to the beneficial effect of OFIF. Interestingly, CrPi also increased the abundance of *NK4A214_group*, suggesting a partial overlap in microbiota modulation between CrPi and OFIF.

In comparison with the HFD group, OFIF treatment reduced the abundance of the *Romboutsia*, *Sporosarcina* and *Lachnoclostridium* genera, the abundance of which was positively correlated with various markers of glucose dysmetabolism, in particular HOMA‐IR, index of IR, oxidative stress and inflammation. Our observations are consistent with previous reports that highlight the pathogenic nature of *Romboutsia*,^54^ *Lachnoclostridium* ^55^ and *Sporosarcina*,^56^ all of which are associated with obesity, inflammation and type 2 diabetes. Interestingly, the use of metformin is also associated with reduced *Romboutsia* abundance in the gut microbiota.^57^ CrPi supplementation also reduced the abundance of *Romboutsia*, suggesting a shared protective effect with OFIF in modulating this potentially harmful genus. Additionally, the reduction in the abundance of *Staphylococcus* and *Enterorhabdus* genera observed in OFIF‐supplemented mice could represent also a healthy advantageous action. In fact, our Spearman analysis indicated a positive correlation between *Staphylococcus* and all parameters of oxidative stress and inflammation we considered. In line with our results, *Staphylococcus* has been reported to be associated with chronic inflammation and IR.^51^ In our analysis, *Enterorhabdus* positively correlated with the parameters of glucose dysmetabolism and some inflammatory markers in accord with other reports showing a positive correlation with fasting blood glucose levels, HbA1c% and HOMA‐IR.^58^ Indeed, the *Enterorhabdus* genus has been associated with various health issues, including type 2 diabetes, obesity and intestinal inflammatory disorders in both humans and mice.^59^ Lastly, CrPi uniquely increased other potentially beneficial genera that were not affected by OFIF, such as *Candidatus Arthromitus*, *Marvinbryantia* and *Lachnospiraceae_UCG‐006*. Notably, *Marvinbryantia and Lachnospiraceae_UCG‐006* are SCFA‐producing bacteria^60^ and *Candidatus Arthromitus* is a symbiotic bacterium involved in immune response.^61^ Conversely, CrPi uniquely reduced harmful genera such as *Acetatifactor*, which is positively associated with HFD‐induced inflammation,^62^ and *Blautia*, elevated levels of which have been involved in the development of glucose metabolism disorders.^63^

## CONCLUSIONS

The present study highlights the multifaceted potential of OFIF in counteracting obesity‐induced, oxy‐inflammation‐related IR, with an efficacy similar to that of CrPi. Beyond its antioxidative, anti‐inflammatory and euglycemic properties, the ability of OFIF to positively modulate gut microbiota composition emerges as a hypothetical mechanism underlying its metabolic benefits.

## AUTHOR CONTRIBUTIONS

**Simona Terzo**: Methodology, Investigation, Data curation, Conceptualisation, Writing. **Pasquale Calvi:** Methodology, Investigation, Data analysis, Conceptualisation and writing. **Ignazio Restivo:** Methodology, Investigation. **Alessandro Massaro**: Methodology, Investigation. **Luisa Tesoriere:** Supervision. **José‐Manuel Fernández‐Real:** Writing – review and editing. **Marta Giardina**: Methodology, Investigation. **Antonella Amato**: Conceptualisation, Supervision. **Flavia Mulè**: Funding acquisition, Conceptualisation, Writing – review & editing, Supervision. **Mario Allegra**: Conceptualisation, Design of study, Writing.

## Supporting information


**Figure S1.** Volcano plot of differential microbial abundance between STD mice and HFD mice. Significantly different genera are coloured according to phylum. Significance was set at p‐value <0.1.

**Table S1.** Composition and caloric content of standard diet and high fat diet.

## Data Availability

All primary data used to support the findings of this study are included within the article and are available from the corresponding authors upon request.
