# Supplementary material for: Opuntia ficus‐indica fruit consumption improves insulin resistance in mice with diet‐induced obesity
Source: J Sci Food Agric. 2025 Jul 25;105(14):7868–80. doi: 10.1002/jsfa.70038 (PMC12509048; doi:10.1002/jsfa.70038)
Supplement: Supplementary file 1 — Figure S1. Volcano plot of differential microbial abundance between STD mice and HFD mice. Significantly different genera are coloured according to phylum. Significance was set at p‐value <0.1. [file JSFA-105-7868-s002.docx]

Figure 1S. Volcano plot of differential microbial abundance between STD mice and HFD mice. Significantly different genera are coloured according to phylum. Significance was set at p-value <0.1.
