# Supplementary material for: Opuntia ficus‐indica fruit consumption improves insulin resistance in mice with diet‐induced obesity
Source: J Sci Food Agric. 2025 Jul 25;105(14):7868–80. doi: 10.1002/jsfa.70038 (PMC12509048; doi:10.1002/jsfa.70038)
Supplement: Supplementary file 2 — Table S1. Composition and caloric content of standard diet and high fat diet. [file JSFA-105-7868-s001.docx]

**Supplementary Table S1. Composition and caloric content of standard diet and high fat diet**

| g/kg | Standard Diet | High-Fat Diet |
| --- | --- | --- |
| Acid Casein 741 | 200 | 265 |
| L-Cystine | 2.8 | 4 |
| Maltodextrine-0032 | 33.2 | 160 |
| Sucrose | 300 | 90 |
| Cellulose (Arbocel) | 50 | 65.5 |
| Soybean Oil | 25 | 30 |
| Lard | 19 | 220 |
| Vitamin mix | 10 | 21 |
| Mineral mix | 45 | 48 |
| Choline Bitartrate | 1.9 | 3 |
| Calcium Phosphate Dibasic | 13 | 3.4 |
| Protein % | 20 | 20 |
| Carbohydrate % | 70 | 20 |
| Fat % | 10 | 60 |
| Total Energy, Kcal/g | 3.5 | 6 |
